# Quantifying the Public Health Impact of Lyme Disease in Minnesota: A Simulation Analysis of Reported and Unreported Cases

**DOI:** 10.36469/001c.146618

**Published:** 2025-11-20

**Authors:** Kathleen E. Angell, M. Jana Broadhurst, Jianghu J. Dong, Tzeyu L. Michaud, Abraham Degarege, Roberto Cortinas, David M. Brett-Major

**Affiliations:** 1 University of Nebraska Medical Center, Omaha, Nebraska; 2 University of Nebraska, Lincoln, Nebraska

**Keywords:** Lyme disease, economic impact, simulation modeling, disease burden, vector-borne

## Abstract

**Background:** Lyme disease, the most common vector-borne disease in Minnesota, is estimated to be underreported by a factor of 10. Delayed diagnosis and misdiagnosis may lead to health complications and increased personal and societal costs. Environmental factors can help to predict high disease years, allowing for early intervention to decrease disease burden. **Objective:** To estimate the health and cost burdens of Lyme disease and the extent to which they could be diminished by public health intervention when high-incidence Lyme disease years are forecasted. **Methods:** We used 5 two-dimensional Monte Carlo simulations to estimate (1) average annual expected burden of Lyme disease, (2 and 3) average burden in low- and high-incidence years, and (4 and 5) the expected burden saved with public health educational interventions preceding high-incidence years. We employed cases reported to the Minnesota Department of Health adjusted for estimates of underreporting found in the literature. **Results:** Among an average of 8436 Lyme disease cases annually, 6074 of them were unidentified. High-incidence years saw over 3700 more cases than low-incidence years, with incremental costs to patients and society exceeding 3million.Weestimatedthatpublichealtheducationbeforehigh−incidenceyearscouldreduceLymediseasecasesby390to787annually,savingupto1.9 million in societal costs. **Discussion:** The simulations presented revealed substantial health and cost burden from Lyme disease, including hidden impacts from undiagnosed and unreported cases. Burden varied widely between high- and low-incidence years, highlighting the need to prioritize prevention when peak years are predicted. While we estimated the effects of individual prevention measures, real-world interventions often combine strategies, potentially producing a greater, multiplicative impact, suggesting our estimates may be conservative. **Conclusions:** Simulation modeling demonstrates Lyme disease’s significant impact on individuals and society. Annual forecasting-triggered public health interventions could reduce cost and disease burden, and these findings may help justify the cost of prevention efforts in policy decision-making.

## INTRODUCTION

Lyme disease is the most common vector-borne disease reported in Minnesota and the United States, with over 2600 and 63 000 cases reported in 2022, respectively.^1,2^ The US Centers for Disease Control and Prevention (CDC) estimates that case counts are 10 times higher than reported, which is supported by several studies.^3–6^ In Minnesota, a study examining physician reporting found that Lyme disease cases meeting the case definition were underreported by a factor of 2.8.^7^ It is unknown to what extent Lyme disease cases in Minnesota are underdiagnosed or misdiagnosed vs unreported.

Lyme disease is characterized by 3 clinical stages: early localized, early disseminated, and late disseminated. Timely diagnosis and treatment lead to most people recovering within a few weeks.^8,9^ Delays in diagnosis may lead to complications including meningitis, Bell’s palsy, carditis, arthritis, more intensive treatment regimens, and increased costs.^8–10^ Among those treated, 10% will develop posttreatment Lyme disease syndrome (PTLDS), characterized by persistent symptoms lasting 6 months after treatment.^9,11^ Due to nonspecific symptoms in the early localized stage, misdiagnosis (>50% of cases) and delayed treatment (>30%) remain common.^11,12^ The burden of these outcomes on a population level is largely unknown.

Tick abundance and environmental factors are associated with high seasons of human risk for Lyme disease in the same year and up to 2 years in advance.^13–15^ Predictive systems, when used for early warning of high-incidence years, present an opportunity for cost-effective public health intervention through tick bite prevention education. While estimates for the efficacy of prevention measures in reducing Lyme disease exist, their impact on reducing population-level disease burden remains underexplored.^16–19^

Lyme disease incidence from the Minnesota Department of Health (MDH), along with estimated rates for underreporting, adverse outcomes, and efficacy of preventive measures, each can inform models assessing population effects of disease and potential benefits of early warning. The objective of this study was to estimate population health burden, economic cost, and disability-adjusted life-years (DALYs) lost from Lyme disease and the extent to which these outcomes could be diminished by public health education implemented in years when predictive models forecast high Lyme disease incidence.

## METHODS

### Model Description

We used 2-dimensional Monte Carlo simulations to estimate the health and economic burden of Lyme disease in the 7-county Twin Cities metropolitan area of Minnesota, which has a population of over 3 million.^20^ We used the number of Lyme disease cases reported to MDH between 2014 and 2023 and factors affecting the variability of these inputs to obtain estimates of burden. The 2-dimensional approach allows us to account for both variability (naturally occurring heterogeneity) and uncertainty (imprecision around a true value) of factors included in the simulations.

### Model Inputs and Outcomes

Factors affecting simulated Lyme disease case counts and outcomes, input effects used, and their definition are presented in **Table 1**. We calculated the mean and SD for the number of cases reported to MDH between 2014 and 2023 and then multiplied this number by 10, the factor by which Lyme disease is estimated to be underreported nationally, to obtain estimated annual cases for the 7-county metropolitan area.^6^ We multiplied the number of reported cases to MDH by 2.8 to estimate the number of cases that were diagnosed and treated, regardless of their reporting status.^7^ We subtracted the number of clinician-diagnosed cases from the overall estimated cases to determine the number of unidentified cases, either through missed diagnosis, failure to seek care and treatment, or other means.

**Table 1.** Inputs and Outcome Parameters Implemented in the Monte Carlo Lyme Disease Burden Model

**Parameter**	**Definition**	**Input Effect**	**Simulation**	**Dimension**	**Source**
Case counts					
Estimated annual cases	Average cases with underreporting multiplication factor	10×	1-5	Fixed	Cook et al, 2020^3^
Reported cases	Average cases in 7-county metro by year ± SD	n = 883.6 SD = 244.3; normal distribution	1-5	Variability	Annual incidence (2014-2018) provided by MDH
Clinician-diagnosed cases	Reported cases × 2.8 underreporting multiplier	2.8×	1-5	Fixed	Schiffman et al, 2018^7^
Unreported cases	Estimated total clinician diagnosed cases minus reported cases	NA	1-5	NA	NA
Unidentified cases	Estimated annual cases minus clinician diagnosed and treated cases	NA	1-5	NA	NA
Protective clothing	Proportion of cases prevented with use of protective clothing when spending time outdoors	Probability = 0.40 SD = 0.051; normal distribution	4	Uncertainty	Vasquez et al, 2008^20^
Tick repellent	Proportion of cases prevented with use of tick repellent when spending time outdoors	Probability = 0.20 SD = 0.077; normal distribution	5	Uncertainty	Vasquez et al, 2008^20^
Behavior uptake	Proportion of the public expected to uptake personal protective behaviors with educational interventions	Probability = 0.19 SD = 0.006; normal distribution	4-5	Uncertainty	Daltroy et al, 2007^18^
Outcomes					
Delayed treatment	Proportion of patients with >30 days between symptom onset and treatment	Probability = 0.31 SD = 0.017; normal distribution	1-5	Uncertainty	Hirsch et al, 2020^11^
PTLDS	Proportion of patients with persistent symptoms for >6 mo				
Given on-time treatment		Probability = 0.073 SD = 0.011; normal distribution	1-5	Uncertainty	Hirsch et al, 2020^11^
Given delayed treatment		Probability = 0.14 SD = 0.022; normal distribution	1-5	Uncertainty	Hirsch et al, 2020^11^
Cost to patients	Estimated medical, nonmedical, and cost of productivity losses from patient perspective				
Given PTLDS		$5050/person SD = $2972; log-normal distribution	1-5	Variability	Hook et al, 2022,^10^ Adrion et al, 2015^21^
Given no PTLDS, and for unidentified cases		$1252/person SD = $2972; log-normal distribution	1-5	Variability	Hook et al, 2022^10^
Societal medical costs	Total estimated medical costs from community perspective	$1333/person SD = $5690; log-normal distribution	1-5	Variability	Hook et al, 2022^10^
Disability-adjusted life-years (DALYs) lost	Average DALYs lost per case of Lyme disease	1.661	1-5	Fixed	van den Wijngaard et al, 2015^22^
Neuroborreliosis	Proportion of patients experiencing this disorder	Probability = 0.08 SD = 0.007; normal distribution	1-5	Uncertainty	Berglund et al, 2009^23^
Residual neurologic sequelae	Proportion of neuroborreliosis patients reporting residual neurologic symptoms 5 y post treatment	Probability = 0.25 SD = 0.040; normal distribution	1-5	Uncertainty	Berglund et al, 2009^23^
Lyme carditis	Proportion of patients suffering from this condition	Probability = 0.04-0.10; uniform distribution	1-5	Uncertainty	Krause et al, 2013^24^

Abbreviations: NA, not applicable; MDH, Minnesota Department of Health; PTLDS, posttreatment Lyme disease syndrome.

The simulation structure was divided into two parts, one estimating burden for cases that were diagnosed and treated (clinician-identified cases) and the other for unidentified cases. Outcomes estimated for unidentified cases included the number of people with Lyme carditis, neuroborreliosis, residual neurologic sequelae 5 years post treatment for those with a neuroborreliosis diagnosis, DALYs lost, cost to patients (medical, nonmedical, and productivity losses), and societal medical costs (cost of all Lyme disease–related medical services). Outcomes estimated for identified cases were delayed treatment (>30 days from symptom onset to treatment), PTLDS (estimated separately based on timely or delayed treatment), cost to patients (estimated separately based on diagnosis of PTLDS), societal medical costs, DALYs lost, neuroborreliosis, and residual neurologic sequelae 5 years after treatment for those with neuroborreliosis. A normal distribution was used for most outcomes, except for Lyme carditis, which followed a uniform distribution, and patient and societal costs, which were modeled using a log-normal distribution to account for right-skewed data.

Simulation 1 estimated average disease outcomes across all years. Simulation 2 estimated average disease outcomes across only high-incidence Lyme disease years. Simulation 3 estimated average disease outcomes across only low-incidence Lyme disease years. Simulation 4 and 5 estimated average disease outcomes across high-incidence years if educational programs targeting use of protective clothing and tick repellent had been implemented. Based on prior evidence, we assumed that 19% of individuals not practicing preventive behaviors would adopt protective measures following educational interventions^16^ and that protective clothing (simulation 4) and tick repellent (simulation 5) would yield 40% and 20% efficacy in preventing Lyme disease, respectively.^18^

### Data Analysis

**Figure 1.**
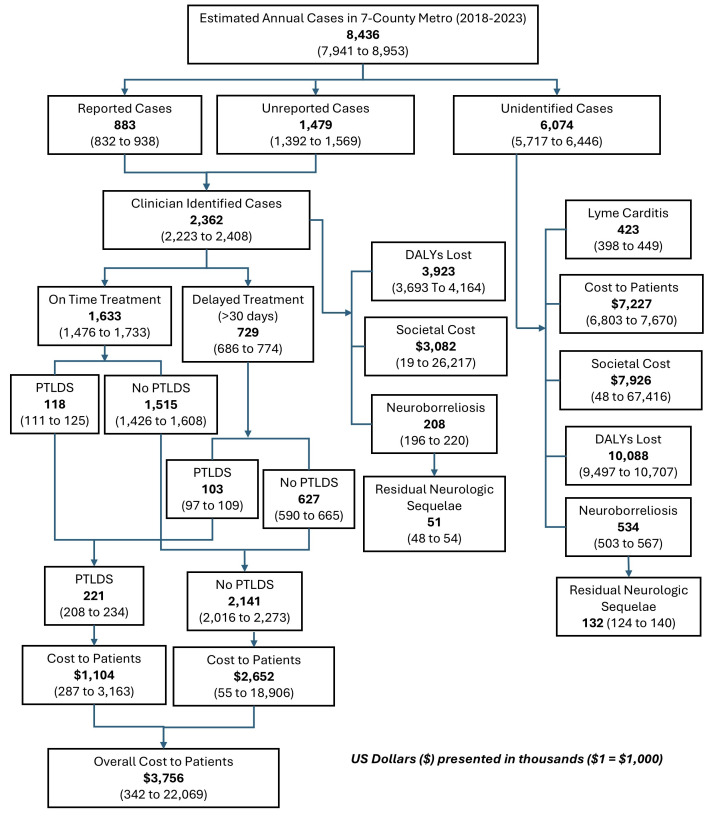
Monte Carlo Simulation 1 Output and Structure (Estimated Average Annual Cases and Outcomes: 2014-2023) Abbreviations: DALY, disability-adjusted life-year; PTLDS, posttreatment Lyme disease syndrome.

All estimation models used the mc2d package in R software, running 1000 iterations each for variability and uncertainty dimensions. R code can be found in the **Supplementary File**. Five sets of burden outcomes were modeled. Simulation 1 estimated the burden of Lyme disease cases reported to MDH, adjusted for underreporting estimations, using data from 2014 to 2023. Simulation 2 provided outcome estimations for years with high Lyme disease incidence, defined as years with case counts above the 50th percentile (2016-2017 and 2022-2023). Simulation 3 provided outcome estimations for years with low Lyme disease incidence, defined as years with case counts at or below the 50th percentile (2014-2015, 2018-2019, and 2021). Simulations 4 and 5 estimated high Lyme disease incidence years adjusting for cases saved from theoretical education programs targeting the use of protective clothing (simulation 4) and tick repellent (simulation 5) when spending time outdoors. **Figure 1** shows a depiction of the simulation structure which flows from estimated annual cases of Lyme disease to estimated burden outcomes. Means and 95% confidence intervals (CI) were provided for the case categories and burden outcomes (**Figure 1)**. Burden estimates from simulations 4 and 5 were subtracted from those in simulation 2 to provide means and 95% CI to estimate burden saved when educational interventions are implemented in high-incidence years.

### Assessing Uncertainty

A sensitivity analysis on overall cost to patients among clinician-identified cases was performed to assess the lability of the modeling from changes in individual input parameters. The analysis was performed on the simulation 1 model for average burden across all years. Input parameters for reported cases, delayed treatment, and PTLDS were independently varied by 1 SD above and below their estimated values (**Table 1**), then entered into the model while holding all other parameters constant. Estimated cost to patients varied by 50% above and below the estimated value due to its distribution.

## RESULTS

Simulation 1 estimated 8436 (95% CI, 7941-8953) annual cases in the Minnesota Twin-Cities metropolitan area (**Figure 1**). Over 2300 estimated cases were diagnosed and treated by a physician with over 6000 unidentified cases. Among clinician-identified cases, we estimated over 700 cases with delay to treatment, 200 cases of PTLDS, 200 cases of neuroborreliosis, 3900 DALYs lost, more than $3.7 million in overall costs to patients, and $3.1 million in societal costs. Among unidentified cases, we estimated over 400 cases of Lyme carditis; 500 cases of neuroborreliosis, including 100 with residual neurologic sequelae; 10 000 DALYs lost; $7.2 million in patient costs; and $7.9 million in societal costs annually.

Case count estimates from simulations 2 and 3 and estimated differences are shown in **Table 2**. On average, high-incidence Lyme disease years (simulation 2) yielded 3704 (1590-5913) more cases than low-incidence years (simulation 3). Among clinician-identified cases, high-incidence years account for 320 more cases of delayed treatment, 97 of PTLDS, 1722 DALYs lost, 91 of neuroborreliosis, over $2.1 million in individual costs and $1.9 million in societal costs. Among the 2667 additional unidentified cases, the simulation estimated 186 more cases of Lyme carditis; 234 of neuroborreliosis, 58 with residual neurologic symptoms; 4429 DALYs lost; $3.2 million in patient costs; and over $4 million in societal costs.

**Table 2.** Simulation 2 and 3 Output: Average Annual Outcomes for High- and Low-Incidence Lyme Disease Years

**Outcome**	**Simulation 2: High-Incidence Years ($)**	**Simulation 3: Low-Incidence Years ($)**	**Difference Between High- and Low-Incidence Years ($)**
Overall cases	10 418 (6314-14 709)	6714 (4723-8796)	3704 (1590-5913)
Clinician-identified cases			
Reported cases	1091 (661-1540)	703 (495-921)	388 (167-619)
Unreported cases	1826 (1107-2578)	1177 (828-1542)	649 (29-1036)
Delayed treatment	901 (546-1272)	580 (408-760)	320 (137-511)
PTLDS			
With delayed treatment	127 (77-179)	82 (58-107)	45 (19-72)
Without delayed treatment	146 (88-206)	94 (66-123)	52 (22-83)
Cost-identified patients			
With PTLDS	$1489 (228-5197)	$936 (171-3108)	$553 (58-2089)
Without PTLDS	$4009 (44-31 061)	$2446 (33-18 575)	$1563 (11-12 486)
Societal medical costs	$4844 (14-43 073)	$2928 (11-25 758)	$1917 (4-17 314)
DALYs lost	4545 (2936-6841)	3123 (2197-4091)	1722 (740-2750)
Neuroborreliosis	257 (155-362)	165 (116-217)	91 (39-146)
Residual neurologic symptoms	63 (38-89)	41 (29-53)	23 (10-36)
Unidentified cases			
All	7501 (4546-10 591)	4834 (3401-6333)	2667 (1145-4257)
Lyme carditis	523 (317-738)	337 (237-441)	186 (80-297)
Neuroborreliosis	660 (400-931)	425 (299-557)	234 (101-374)
Residual neurologic symptoms	163 (99-230)	105 (74-137)	58 (25-92)
DALYs lost	12 459 (7550-17 591)	8030 (5648-10 520)	4429 (1902-7071)
Cost to unidentified patients	$8925 (5409-12 601)	$5752 (4046-7536)	$3173 (1362-5066)
Societal medical costs	$12 458 (38-110 758)	$7529 (29-66 234)	$4929 (10-44 524)

Abbreviations: DALY, disability-adjusted life-year; PTLDS, posttreatment Lyme disease syndrome.

US dollars presented in thousands ($1 = $1000).

Simulations 4 and 5 estimated 9% and 4% of cases, respectively, to be prevented in high-incidence years given educational interventions targeting protective clothing and tick repellent use. Outcomes for simulations 4 and 5 and differences in outcomes can be found in **Table 3**. Educational interventions targeting the use of protective clothing would prevent 787 (95% CI, 477-1111) cases annually in high-incidence years. Additionally, among cases identified by a clinician, 69 cases of delayed treatment, 366 DALYs lost, 21 cases of PTLDS, over $400 000 in individual costs and $300 000 in societal costs would be prevented. Among the 567 unidentified cases that could be prevented, 40 cases of Lyme carditis, 51 cases of neuroborreliosis, 941 DALYs lost, over $800 000 in individual costs, and $900 000 in societal costs could also be prevented. Interventions targeting the use of tick repellent would prevent 390 (95% CI, 236-551) cases per year including 35 cases of delayed treatment, 11 cases of PTLDS, 182 DALYs lost, 10 cases of neuroborreliosis, over $200 000 in individual costs, and $180 000 in societal costs among cases identified by a clinician. Among the 281 preventable unidentified cases 20 cases of Lyme carditis, 26 cases of neuroborreliosis, 467 DALYs lost, $500 000 in individual costs, and $400 000 in societal costs could be prevented.

**Table 3.** Simulations 4 and 5 Output: Average Annual Outcomes for High-Incidence Lyme Disease Years With Projected Cases Saved from Educational Interventions in High-Incidence Years

**Outcome**	**Simulation 4: High Incidence Years With Protective Clothing Preventive Behavior**	**Prevented Cases and Burden in High Incidence Years Due to Protective Clothing**	**Simulation 5: High Incidence Years With Tick Repellent Use Preventive Behavior**	**Prevented Cases and Burden in High Incidence Years Due to Tick Repellent**
Overall cases	9631 (5837-13 598)	787 (477-1111)	10 031 (6077-14 158)	390 (236-551)
Clinician-identified cases				
Reported cases	1009 (611-1424)	82 (50-116)	1050 (636-14 158)	41 (25-58)
Unreported cases	1688 (1023-2383)	138 (84-195)	1758 (1065-2482)	68 (41-97)
Delayed treatment	832 (504-1175)	69 (42-97)	866 (525-1223)	35 (21-49)
PTLDS				
With delayed treatment	117 (71-165)	10 (6-14)	122 (74-172)	5 (3-7)
Without delayed treatment	135 (82-190)	11 (7-16)	140 (85-198)	6 (3-8)
Cost to identified patients				
With PTLDS	1374(211-$4793)	116(18-$403)	1430(219-$4988)	60(9-$209)
Without PTLDS	3707(40-$28 721)	302(3-$2340)	3860(42-$29 906)	149(1-$1156)
Societal medical costs	4479(14-$39 818)	366(1-$3254)	4663(14-$41 459)	182(-8-$1615)
DALYs lost	4479 (2714-6324)	366 (222-517)	4664 (2826-6585)	182 (110-256)
Neuroborreliosis	237 (144-334)	20 (12-28)	247 (149-348)	10 (6-14)
Residual neurologic symptoms	58 (35-82)	5 (3-7)	61 (37-86)	3 (2-4)
Unidentified cases				
All	6934 (4202-9790)	567 (343-800)	7220 (4375-10 194)	281 (170-397)
Lyme carditis	483 (293-682)	40 (24-56)	503 (305-710)	20 (12-28)
Neuroborreliosis	609 (369-860)	51 (31-71)	634 (384-895)	26 (16-36)
Residual neurologic symptoms	150 (91-212)	13 (8-18)	156 (95-220)	7 (4-10)
DALYs lost	11 518 (6980-16 262)	941 (570-1329)	11 992 (7268-16 932)	467 (283-659)
Cost to unidentified patients	$8096 (4906-11 430)	$829 (503-1171)	$8386 (5082-11 841)	$538 (326-760)
Societal medical costs	11 517(35-$102 390)	941(3-$8368)	11 992(37-$106 608)	467(-1 to $4152)

Abbreviations: DALY, disability-adjusted life-year; PTLDS, posttreatment Lyme disease syndrome.

US dollars presented in thousands ($1 = $1000).

**Figure 2.**
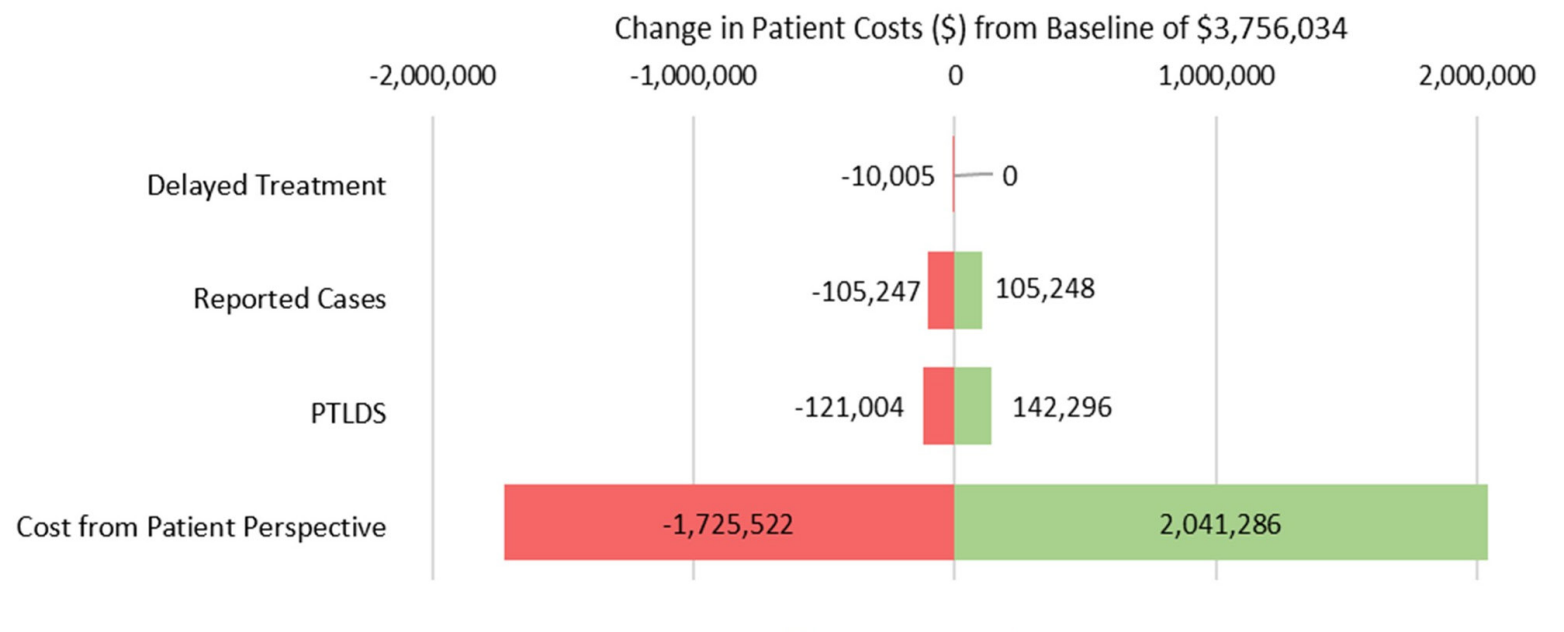
Sensitivity of Patient Costs to Changes in Individual Parameters Abbreviation: PTLDS, posttreatment Lyme disease syndrome. Parameter variation by 1 SD above and below the mean, except for estimated cost to patients which varied by 50%.

In sensitivity analysis, preventable patient costs remained apparent even with large changes in input parameters. **Figure 2** summarizes the impact of changes to parameters on overall cost to patients for the simulation 1 model. Estimates for cost have the highest impact on variation in the outcome, changing the simulated estimate by up to $2 million in either direction. Varying the estimate for delay to treatment has the lowest impact on overall cost to patients, changing the simulated estimate by up to $10 000. Varying the estimates for reported cases and PTLDS changes the estimate by about $100 000 and $130 000, respectively.

## DISCUSSION

This simulation, the first of its kind in Minnesota, presents substantial health and cost burden attributable to Lyme disease. In addition to high cost and DALYs lost, these outcomes depict the hidden burden of infected individuals who remain unidentified, reflecting the impact of underreporting and underdiagnosis. Simulations 4 and 5 reflect the burden and cost savings that could be attained in high-incidence years when tick bite prevention education is implemented. Given the substantial differences in burden and cost between high- and low-incidence years, implementing strategies to mitigate incidence in the high-incidence years should be a priority.

Simulation 1 showed a significant population-level health burden, particularly given estimates for underreporting, which identified over 1400 clinician-diagnosed cases unreported and 6000 unidentified cases. These results were particularly staggering when we explored the downstream estimates, such as over 200 PTLDS cases, $10 million in patient costs, and 14 000 DALYs lost. One study found the estimated cost per DALY to be $69 499, making estimates for DALYs lost and the actual cost to society even more staggering.^25^ These estimates uncover the known (reported cases) and unknown (unreported and unidentified) impacts of Lyme disease in the population.

Heightened average case numbers and health burdens in high-incidence Lyme disease years presents an opportunity for public health intervention, particularly given that high-incidence years can be predicted through environmental factors as far as 2 years in advance and through tick abundance measures in the same year.^19^ Simulations 4 and 5 show an estimated 9% (787) and 4% (390) of cases can be prevented annually in high-incidence years through tick bite prevention interventions. While these numbers may seem small given the overall burden of disease, they represent real savings in health outcomes, DALYs lost, and cost. Additionally, while this study estimates the impact of tick bite prevention measures individually, real-world educational interventions typically promote multiple preventive strategies. This could further enhance case reductions, potentially yielding a multiplicative effect when measures are combined.

We introduced an estimate to account for the proportion of people above the baseline expected to adopt preventive behavior after educational intervention. In the source used, 39% of individuals were already practicing preventive behavior and an additional 19% started the behavior after education.^16^ This estimate (19%) highlights the need for further research to optimize behavior change interventions for vector-borne disease prevention, with the goal of increasing the proportion of individuals who adopt protective behaviors. In this study, we simulated the burden saved from interventional programs in high-disease-incidence years as opposed to all years. Evidence exists suggesting chronic message fatigue, where populations are exposed to similar risk communication messaging over a prolonged period, induces a state of resistance to health behaviors in the population.^26^ Implementing tick bite prevention interventions beyond normal messaging in high-incidence years allows for high impact while avoiding message fatigue.

The sensitivity analysis suggests the model to be robust when exploring overall cost to patients, the most downstream outcome in the model structure. Given the wide CI for the cost parameter input, it is unsurprising that changing this estimate has the largest effect on the simulated value. Variation in the delay to treatment estimate has the smallest effect on patient costs, probably due to relatively narrow confidence limits of the estimate. Findings from this analysis suggest that the overall impact of a parameter estimate is primarily influenced by two factors: its position within the model hierarchy and the breadth of its confidence interval. PTLDS does not have a particularly wide CI, but is the second most influential parameter, likely due to its position in the model structure and it’s downstream effects on cost.

Wide CIs in cost estimates reflect uncertainty in the data, which likely arises from the variability in patient experiences, ranging from early identification and treatment with lower costs to delayed diagnosis associated with more severe symptoms, complex treatment and higher costs to both patients and society. Patient cost estimates used considered insurance status of individuals; 70% of study participants had private insurance, likely deflating this cost estimate.^10^ Individuals without private insurance and uninsured individuals likely bear the highest burden of cost, presenting a barrier to access to care. While insurance status was not factored into our simulation estimates, those who are uninsured or underinsured are more likely to delay or not seek medical care at all, putting this group at enhanced risk for late-disseminated disease and more severe symptoms.^27^ Public health interventions should address this gap in health equity through education on early symptoms of Lyme disease for high-risk groups, as well as resources for testing and treatment among uninsured and underinsured groups.

The unidentified cases in the simulation structure presents potential error and misclassification bias. These cases may be unidentified due to missed diagnosis by a physician, failure to seek care, either due to access barriers or mild to asymptomatic presentation. Alternatively, perhaps the patient was correctly diagnosed and treated but not reported or documented appropriately. Although the CDC and others estimate a 10-fold underestimation of Lyme disease cases, it is possible this is more applicable to the endemic Northeast rather than the Midwest. Additionally, we estimated unreported but diagnosed and treated cases with a multiplication factor of 2.8, identified by a study that took place in an endemic county in Minnesota outside the metropolitan area. It is possible this estimate is not accurate for the 7-county metropolitan area. If the CDC underestimation factor is inflated, or the Minnesota underreporting factor is deflated, the numbers in the unidentified category would decrease. As the unidentified cases in this study are largely an unknown entity, we erred toward conservative estimates, including inputs for the general Lyme disease population for outcomes such as DALYs, cost, and neuroborreliosis. Incidence of Lyme carditis was estimated for the unidentified cases only as the condition is associated with chronically untreated Lyme disease, making the condition extremely rare among those diagnosed and treated.^28^ Given gaps in knowledge associated with the unidentified cases, it is unknown whether the range used to estimate carditis is an overestimation or an underestimation.

One strength of this study is its integration of estimates from a variety of sources to comprehensively assess the burden of Lyme disease in an understudied Lyme-endemic region of the country. While several studies can be found estimating the burden of specific Lyme disease outcomes in the United States, there is a paucity of those estimating burden on the population level with adjustments for underreporting rates.^21–23,29,30^ Additionally, the use of two-dimensional Monte Carlo simulation allowed us to account for both variability and uncertainty within the model structure. This method allowed us to simulate a variety of burden outcomes, nested within each other, allowing the error at one level to impact the outcome of others. One limitation is the societal cost simulated considers all medical costs regardless of the payer but does not account for indirect costs such as productivity losses. Another limitation of this study is the use of estimates from secondary sources, which may not always be generalizable to the population of the Minnesota 7-county Twin Cities metropolitan area. Although we expected estimates to vary to some degree geographically, the incorporation of error estimates helped to account for the variation expected in our population. Some studies have noted differences between the Midwest and Northeast in the environmental factors that impact tick abundance and risk of Lyme disease based on vector infection, emphasizing the importance that this work be conducted in different regions in order to gain a more accurate national perspective.^31,32^

## CONCLUSIONS

Lyme disease presents significant public health burden with high individual and societal costs, particularly in high-incidence years. Underreporting, underdiagnosis, and misdiagnosis of Lyme disease lead to uncertainty regarding true burden, although our simulations showed that over 6000 cases are unaccounted annually in Minnesota alone. Given the ability to forecast high-incidence years, implementing tick bite prevention education in anticipation of these years is a logical and proactive public health strategy. Simulation results presented here show substantial economic savings that could outweigh the cost of implementation.

## Supplementary Material

Online Supplementary Material R Code for Estimation Models
