# Supplementary material for: Quantifying the Public Health Impact of Lyme Disease in Minnesota: A Simulation Analysis of Reported and Unreported Cases
Source: J Health Econ Outcomes Res. 2025 Nov 20;12(2):221–8. doi: 10.36469/001c.146618 (PMC12640196; doi:10.36469/001c.146618)
Supplement: Online Supplementary Material — R Code for Estimation Models [file jheor_2025_12_2_146618_310371.docx]

library(mc2d)

ndvar(1000)

ndunc(1000)

options(scipen=999)

############################################################

################ All years (2014-2023) ###################

############################################################

### For the Misdiagnosis Side of the Map ###

Cases <- mcstoc(rnorm, type="V", seed=6824, mean=8451, sd=244.3)

MisDx <- Cases*.72

CarditisRate <- mcstoc(runif, type="U", seed=6824, min=0.04, max=0.10)

CostRate <- mcstoc(rlnorm, type="U", seed=6824, mean=6.19, sd=1.38)

NeuroRate <- mcstoc(rnorm, type="U", seed=6824, mean=0.0884, sd=0.00740)

ResidNeuroRate <- mcstoc(rnorm, type="U", seed=6824, mean=.2456, sd=.0403)

SocietyCostRate <- mcstoc(rlnorm, type="V", seed=6824, mean=5.72, sd=1.72)

Carditis <- MisDx*CarditisRate

Cost <- MisDx*CostRate

SocietyCost <- MisDx*SocietyCostRate

DALYs <- MisDx*1.661

Neuroborrel <- MisDx*NeuroRate

ResidNeuro <-Neuroborrel*ResidNeuroRate

MisDxResults <- mc(Cases, MisDx, Carditis, Cost, DALYs, Neuroborrel, ResidNeuro,

SocietyCost)

print (MisDxResults, digits=10)

summary(MisDxResults)

### For the Diagnosed and Treated Side of the Map ###

#Estimate the Diagnosed Cases

Cases <- mcstoc(rnorm, type="V", seed=6824, mean=8451, sd=244.3)

Diagnosed <- Cases*.28

Reported <- Diagnosed*.374

unreported <- Diagnosed - Reported

#Estimate Neuroborreliosis and residual Neuro

NeuroRate <- mcstoc(rnorm, type="U", seed=6824, mean=0.0884, sd=0.00740)

ResidNeuroRate <- mcstoc(rnorm, type="U", seed=6824, mean=.2456, sd=.0403)

Neuroborrel <- Diagnosed*NeuroRate

ResidNeuro <-Neuroborrel*ResidNeuroRate

#Estimate DALYS

DALYs <- Diagnosed*1.661

#Estimate delayed and on time treatment

DelayRate <- mcstoc(rnorm, type="U", seed=6824, mean=.3098, sd=.01658)

DelayedTreat <- Diagnosed*DelayRate

OnTimeTreat <- Diagnosed-DelayedTreat

##For On Time Treatment##

#Estimate PTLD and No PTLD

PTLDonTimeRate <- mcstoc(rnorm, type="U", seed=6824, mean=0.0733, sd=0.01125)

PTLDonTime <- OnTimeTreat*PTLDonTimeRate

noPTLDonTime <- OnTimeTreat-PTLDonTime

##For Delay Treatment##

#Estimate PTLD and No PTLD

PTLDDelayRate <- mcstoc(rnorm, type="U", seed=6824, mean=0.141, sd=0.0224)

PTLDDelay <- DelayedTreat*PTLDDelayRate

noPTLDDelay <- DelayedTreat-PTLDDelay

#Add the PDLT yes and no groups Together

PTLD <- PTLDonTime+PTLDDelay

NoPTLD <- noPTLDonTime + noPTLDDelay

#Cost for the PTLD Group.

PTLDCostRate <- mcstoc(rlnorm, type="V", seed=6824, mean=8.38, sd=0.55)

PTLDCost <- PTLD*PTLDCostRate

#Cost for the non PTLD Group

NoPTLDCostRate <- mcstoc(rlnorm, type="V", seed=6824, mean=6.19, sd=1.38)

NoPTLDCost <- NoPTLD*NoPTLDCostRate

TotalCost <- PTLDCost+NoPTLDCost

#Societal Cost

SocietyCostRate <- mcstoc(rlnorm, type="V", seed=6824, mean=5.72, sd=1.72)

SocietyCostDx <- Diagnosed*SocietyCostRate

#Finish it up#

DxResults <- mc(Cases, Diagnosed, Reported, unreported, Neuroborrel, ResidNeuro, DALYs, DelayedTreat,

OnTimeTreat, PTLDonTime, noPTLDonTime, PTLDDelay, noPTLDDelay,

PTLD, NoPTLD, PTLDCost, NoPTLDCost, TotalCost, SocietyCostDx)

print (DxResults, digits=3)

summary(DxResults)

###############################################################

####### Above Median Years Only (2016, 17, 22, 23) ##########

###############################################################

### For the Misdiagnosis Side of the Map ###

HiCases <- mcstoc(rnorm, type="V", seed=6824, mean=10545, sd=2026)

HiMisDx <- HiCases*.72

HiCarditisRate <- mcstoc(runif, type="U", seed=6824, min=0.04, max=0.10)

HiCostRate <- mcstoc(rlnorm, type="U", seed=6824, mean=6.19, sd=1.38)

HiNeuroRate <- mcstoc(rnorm, type="U", seed=6824, mean=0.0884, sd=0.00740)

HiResidNeuroRate <- mcstoc(rnorm, type="U", seed=6824, mean=.2456, sd=.0403)

SocietyCostRate <- mcstoc(rlnorm, type="V", seed=6824, mean=5.72, sd=1.72)

HiSocietyCost <- HiMisDx*SocietyCostRate

HiCarditis <- HiMisDx*HiCarditisRate

HiCost <- HiMisDx*HiCostRate

HiDALYs <- HiMisDx*1.661

HiNeuroborrel <- HiMisDx*HiNeuroRate

HiResidNeuro <-HiNeuroborrel*HiResidNeuroRate

HiMisDxResults <- mc(HiCases, HiMisDx, HiCarditis, HiCost, HiDALYs,

HiNeuroborrel, HiResidNeuro, HiSocietyCost)

print (HiMisDxResults, digits=5)

summary(HiMisDxResults)

### For the Diagnosed and Treated Side of the Map ###

#Estimate the Diagnosed Cases

HiDCases <- mcstoc(rnorm, type="V", seed=6824, mean=10545, sd=2026)

HiDDiagnosed <- HiDCases*.28

HiReported <- HiDDiagnosed*.374

Hiunreported <- HiDDiagnosed - HiReported

#Estimate Neuroborreliosis and residual Neuro

HiDNeuroRate <- mcstoc(rnorm, type="U", seed=6824, mean=0.0884, sd=0.00740)

HiDResidNeuroRate <- mcstoc(rnorm, type="U", seed=6824, mean=.2456, sd=.0403)

HiDNeuroborrel <- HiDDiagnosed*HiDNeuroRate

HiDResidNeuro <-HiDNeuroborrel*HiDResidNeuroRate

#Estimate DALYS

HiDDALYs <- HiDDiagnosed*1.661

#Estimate delayed and on time treatment

HiDDelayRate <- mcstoc(rnorm, type="U", seed=6824, mean=.3098, sd=.01658)

HiDDelayedTreat <- HiDDiagnosed*HiDDelayRate

HiDOnTimeTreat <- HiDDiagnosed-HiDDelayedTreat

##For On Time Treatment##

#Estimate PTLD and No PTLD

HiDPTLDonTimeRate <- mcstoc(rnorm, type="U", seed=6824, mean=0.0733, sd=0.01125)

HiDPTLDonTime <- HiDOnTimeTreat*HiDPTLDonTimeRate

HiDnoPTLDonTime <- HiDOnTimeTreat-HiDPTLDonTime

##For Delay Treatment##

#Estimate PTLD and No PTLD

HiDPTLDDelayRate <- mcstoc(rnorm, type="U", seed=6824, mean=0.141, sd=0.0224)

HiDPTLDDelay <- HiDDelayedTreat*HiDPTLDDelayRate

HiDnoPTLDDelay <- HiDDelayedTreat-HiDPTLDDelay

#Add the PDLT yes and no groups Together

HiDPTLD <- HiDPTLDonTime+HiDPTLDDelay

HiDNoPTLD <- HiDnoPTLDonTime + HiDnoPTLDDelay

#Cost for the PTLD Group.

HiDPTLDCostRate <- mcstoc(rlnorm, type="V", seed=6824, mean=8.38, sd=0.55)

HiDPTLDCost <- HiDPTLD*HiDPTLDCostRate

#Cost for the non PTLD Group

HiDNoPTLDCostRate <- mcstoc(rlnorm, type="V", seed=6824, mean=6.19, sd=1.38)

HiDNoPTLDCost <- HiDNoPTLD*HiDNoPTLDCostRate

#Societal Cost

SocietyCostRate <- mcstoc(rlnorm, type="V", seed=6824, mean=5.72, sd=1.72)

HiDSocietyCost <- HiDDiagnosed*SocietyCostRate

#Finish it up#

HiDDxResults <- mc(HiDCases, HiDDiagnosed, HiReported, Hiunreported, HiDNeuroborrel, HiDResidNeuro,

HiDDALYs, HiDDelayedTreat, HiDOnTimeTreat, HiDPTLDonTime,

HiDnoPTLDonTime, HiDPTLDDelay, HiDnoPTLDDelay, HiDPTLD,

HiDNoPTLD, HiDPTLDCost, HiDNoPTLDCost, HiDSocietyCost)

print (HiDDxResults, digits=3)

summary(HiDDxResults)

###################################################################

####### Above Median Years: Use of Protective Clothing ##########

###################################################################

### For the Misdiagnosis Side of the Map ###

Cases <- mcstoc(rnorm, type="V", seed=6824, mean=10545, sd=2026)

ClothesProtectRate <- mcstoc(rnorm, type="U", seed=6824, mean=0.4, sd=0.051)

BehaviorUptakeRate <- mcstoc(rnorm, type="U", seed=6824, mean=0.19, sd=0.00568)

ClotheXBehaviorUptake <- ClothesProtectRate*BehaviorUptakeRate

ClotheXBehaviorUptakeRate <- 1-ClotheXBehaviorUptake

ClothesProtectCases <- Cases*ClotheXBehaviorUptakeRate

ClothMisDx <- ClothesProtectCases*.72

ClothCarditisRate <- mcstoc(runif, type="U", seed=6824, min=0.04, max=0.10)

ClothCostRate <- mcstoc(rlnorm, type="U", seed=6824, mean=6.19, sd=1.38)

ClothNeuroRate <- mcstoc(rnorm, type="U", seed=6824, mean=0.0884, sd=0.00740)

ClothResidNeuroRate <- mcstoc(rnorm, type="U", seed=6824, mean=.2456, sd=.0403)

SocietyCostRate <- mcstoc(rlnorm, type="V", seed=6824, mean=5.72, sd=1.72)

ClothSocietyCost <- ClothMisDx*SocietyCostRate

ClothCarditis <- ClothMisDx*ClothCarditisRate

ClothCost <- ClothMisDx*ClothCostRate

ClothDALYs <- ClothMisDx*1.661

ClothNeuroborrel <- ClothMisDx*ClothNeuroRate

ClothResidNeuro <-ClothNeuroborrel*ClothResidNeuroRate

ClothMisDxResults <- mc(Cases, ClothMisDx, ClothCarditis, ClothCost,

ClothDALYs, ClothNeuroborrel, ClothResidNeuro,

ClothesProtectRate, ClothesProtectCases, ClotheXBehaviorUptakeRate,

ClothSocietyCost)

print (ClothMisDxResults, digits=11)

summary(ClothMisDxResults, digits=11)

### For the Diagnosed and Treated Side of the Map ###

#Estimate the Diagnosed Cases

Cases <- mcstoc(rnorm, type="V", seed=6824, mean=10545, sd=2026)

ClothesProtectRate <- mcstoc(rnorm, type="U", seed=6824, mean=0.4, sd=0.051)

BehaviorUptakeRate <- mcstoc(rnorm, type="U", seed=6824, mean=0.19, sd=0.00568)

ClotheXBehaviorUptake <- ClothesProtectRate*BehaviorUptakeRate

ClotheXBehaviorUptakeRate <- 1-ClotheXBehaviorUptake

ClothesProtectCases <- Cases*ClotheXBehaviorUptakeRate

ClothesDiagnosed <- ClothesProtectCases*.28

ClothesReported <- ClothesDiagnosed*.374

Clothesunreported <- ClothesDiagnosed - ClothesReported

#Estimate Neuroborreliosis and residual Neuro

ClothDNeuroRate <- mcstoc(rnorm, type="U", seed=6824, mean=0.0884, sd=0.00740)

ClothDResidNeuroRate <- mcstoc(rnorm, type="U", seed=6824, mean=.2456, sd=.0403)

ClothDNeuroborrel <- ClothesDiagnosed*ClothDNeuroRate

ClothDResidNeuro <-ClothDNeuroborrel*ClothDResidNeuroRate

#Estimate DALYS

ClothDDALYs <- ClothesDiagnosed*1.661

#Estimate delayed and on time treatment

ClothDDelayRate <- mcstoc(rnorm, type="U", seed=6824, mean=.3098, sd=.01658)

ClothDDelayedTreat <- ClothesDiagnosed*ClothDDelayRate

ClothDOnTimeTreat <- ClothesDiagnosed-ClothDDelayedTreat

##For On Time Treatment##

#Estimate PTLD and No PTLD

ClothDPTLDonTimeRate <- mcstoc(rnorm, type="U", seed=6824, mean=0.0733, sd=0.01125)

ClothDPTLDonTime <- ClothDOnTimeTreat*ClothDPTLDonTimeRate

ClothDnoPTLDonTime <- ClothDOnTimeTreat-ClothDPTLDonTime

##For Delay Treatment##

#Estimate PTLD and No PTLD

ClothDPTLDDelayRate <- mcstoc(rnorm, type="U", seed=6824, mean=0.141, sd=0.0224)

ClothDPTLDDelay <- ClothDDelayedTreat*ClothDPTLDDelayRate

ClothDnoPTLDDelay <- ClothDDelayedTreat-ClothDPTLDDelay

#Add the PDLT yes and no groups Together

ClothDPTLD <- ClothDPTLDonTime+ClothDPTLDDelay

ClothDNoPTLD <- ClothDnoPTLDonTime + ClothDnoPTLDDelay

#Cost for the PTLD Group.

ClothDPTLDCostRate <- mcstoc(rlnorm, type="V", seed=6824, mean=8.38, sd=0.55)

ClothDPTLDCost <- ClothDPTLD*ClothDPTLDCostRate

#Cost for the non PTLD Group

ClothDNoPTLDCostRate <- mcstoc(rlnorm, type="V", seed=6824, mean=6.19, sd=1.38)

ClothDNoPTLDCost <- ClothDNoPTLD*ClothDNoPTLDCostRate

#Societal cost

SocietyCostRate <- mcstoc(rlnorm, type="V", seed=6824, mean=5.72, sd=1.72)

ClothDSocietyCost <-ClothesDiagnosed*SocietyCostRate

#Finish it up#

ClothDDxResults <- mc(Cases, ClothesDiagnosed, ClothesReported, Clothesunreported, ClothDNeuroborrel, ClothDResidNeuro,

ClothDDALYs, ClothDDelayedTreat, ClothDOnTimeTreat,

ClothDPTLDonTime, ClothDnoPTLDonTime, ClothDPTLDDelay,

ClothDnoPTLDDelay, ClothDPTLD, ClothDNoPTLD, ClothDPTLDCost,

ClothDNoPTLDCost, ClothesProtectRate, ClothesProtectCases, ClothDSocietyCost)

print (ClothDDxResults, digits=3)

summary(ClothDDxResults)

### Cases and outcomes saved with use of protective clothing###

# For Misdiagnosed #

ClothCaseSave <- HiCases - ClothesProtectCases

ClothMisSave <- HiMisDx - ClothMisDx

ClothMisCard <- HiCarditis - ClothCarditis

ClothMisCost <- HiCost - ClothCost

ClothMisDALY <- HiDALYs - ClothDALYs

ClothMisNeuro <- HiNeuroborrel - ClothNeuroborrel

ClothMisNeurResid <- HiResidNeuro - ClothResidNeuro

ClothMisSocietySave <- HiSocietyCost - ClothSocietyCost

# For Diagnosed #

ClothReportedSave <- HiReported - ClothesReported

ClothunreportedSave <- Hiunreported - Clothesunreported

ClothDxSave <- HiDDiagnosed - ClothesDiagnosed

ClothDxNeuro <- HiDNeuroborrel - ClothDNeuroborrel

ClothDxNeurResid <- HiDResidNeuro - ClothDResidNeuro

ClothDxDALY <- HiDDALYs - ClothDDALYs

ClothDxDelay <- HiDDelayedTreat - ClothDDelayedTreat

ClothDxTimelyTreat <- HiDOnTimeTreat - ClothDOnTimeTreat

ClothDxPTLDonTime <- HiDPTLDonTime - ClothDPTLDonTime

ClothDxNoPTLDonTime <- HiDnoPTLDonTime - ClothDnoPTLDonTime

ClothDxPTLDDelay <- HiDPTLDDelay - ClothDPTLDDelay

ClothDxNoPTLDDelay <- HiDnoPTLDDelay - ClothDnoPTLDDelay

ClothDXPTLDTotSave <- HiDPTLD - ClothDPTLD

ClothDxNoPTLDTotSave <- HiDNoPTLD - ClothDNoPTLD

ClothDxPTLDCostSave <- HiDPTLDCost - ClothDPTLDCost

ClothDxNoPTLDCostSave <- HiDNoPTLDCost - ClothDNoPTLDCost

ClothDxSocietySave <- HiDSocietyCost - ClothDSocietyCost

ClothSaveResult <- mc(ClothCaseSave, ClothMisSave, ClothMisCard, ClothMisCost,

ClothMisDALY, ClothMisNeuro, ClothMisNeurResid, ClothDxSave,

ClothDxNeuro, ClothDxNeurResid, ClothDxDALY, ClothDxDelay,

ClothDxTimelyTreat, ClothDxPTLDonTime, ClothDxNoPTLDonTime,

ClothDxPTLDDelay, ClothDxNoPTLDDelay, ClothDXPTLDTotSave,

ClothDxNoPTLDTotSave, ClothDxPTLDCostSave, ClothDxNoPTLDCostSave,

ClothReportedSave, ClothunreportedSave, ClothMisSocietySave,

ClothDxSocietySave)

print (ClothSaveResult, digits=3)

summary(ClothSaveResult)

##############################################################

####### Above Median Years: Use of Tick Repellent ##########

##############################################################

### For the Misdiagnosis Side of the Map ###

Cases <- mcstoc(rnorm, type="V", seed=6824, mean=10545, sd=2026)

RepelProtectRate <- mcstoc(rnorm, type="U", seed=6824, mean=0.2, sd=0.0765)

BehaviorUptakeRate <- mcstoc(rnorm, type="U", seed=6824, mean=0.19, sd=0.00568)

RepelXBehaviorUptake <- RepelProtectRate*BehaviorUptakeRate

RepelxBehaviorUptakeRate <- 1- RepelXBehaviorUptake

RepelProtectCases <- Cases*RepelxBehaviorUptakeRate

RepMisDx <- RepelProtectCases*.72

RepCarditisRate <- mcstoc(runif, type="U", seed=6824, min=0.04, max=0.10)

RepCostRate <- mcstoc(rlnorm, type="U", seed=6824, mean=6.19, sd=1.38)

RepNeuroRate <- mcstoc(rnorm, type="U", seed=6824, mean=0.0884, sd=0.00740)

RepResidNeuroRate <- mcstoc(rnorm, type="U", seed=6824, mean=.2456, sd=.0403)

SocietyCostRate <- mcstoc(rlnorm, type="V", seed=6824, mean=5.72, sd=1.72)

RepSocietyCost <- RepMisDx*SocietyCostRate

RepCarditis <- RepMisDx*RepCarditisRate

RepCost <- RepMisDx*RepCostRate

RepDALYs <- RepMisDx*1.661

RepNeuroborrel <- RepMisDx*RepNeuroRate

RepResidNeuro <-RepNeuroborrel*RepResidNeuroRate

RepMisDxResults <- mc(Cases, RepMisDx, RepCarditis, RepCost, RepDALYs,

RepNeuroborrel, RepResidNeuro, RepelProtectRate,

RepelProtectCases, RepelXBehaviorUptake, RepSocietyCost)

print (RepMisDxResults, digits=3)

summary(RepMisDxResults)

### For the Diagnosed and Treated Side of the Map ###

#Estimate the Diagnosed Cases

Cases <- mcstoc(rnorm, type="V", seed=6824, mean=10545, sd=2026)

RepelProtectRate <- mcstoc(rnorm, type="U", seed=6824, mean=0.2, sd=0.0765)

BehaviorUptakeRate <- mcstoc(rnorm, type="U", seed=6824, mean=0.19, sd=0.00568)

RepelXBehaviorUptake <- RepelProtectRate*BehaviorUptakeRate

RepelxBehaviorUptakeRate <- 1- RepelXBehaviorUptake

RepelProtectCases <- Cases*RepelxBehaviorUptakeRate

RepDDiagnosed <- RepelProtectCases*.28

RepDReported <- RepDDiagnosed*.374

RepDunreported <- RepDDiagnosed - RepDReported

#Estimate Neuroborreliosis and residual Neuro

RepDNeuroRate <- mcstoc(rnorm, type="U", seed=6824, mean=0.0884, sd=0.00740)

RepDResidNeuroRate <- mcstoc(rnorm, type="U", seed=6824, mean=.2456, sd=.0403)

RepDNeuroborrel <- RepDDiagnosed*RepDNeuroRate

RepDResidNeuro <-RepDNeuroborrel*RepDResidNeuroRate

#Estimate DALYS

RepDDALYs <- RepDDiagnosed*1.661

#Estimate delayed and on time treatment

RepDDelayRate <- mcstoc(rnorm, type="U", seed=6824, mean=.3098, sd=.01658)

RepDDelayedTreat <- RepDDiagnosed*RepDDelayRate

RepDOnTimeTreat <- RepDDiagnosed-RepDDelayedTreat

##For On Time Treatment##

#Estimate PTLD and No PTLD

RepDPTLDonTimeRate <- mcstoc(rnorm, type="U", seed=6824, mean=0.0733, sd=0.01125)

RepDPTLDonTime <- RepDOnTimeTreat*RepDPTLDonTimeRate

RepDnoPTLDonTime <- RepDOnTimeTreat-RepDPTLDonTime

##For Delay Treatment##

#Estimate PTLD and No PTLD

RepDPTLDDelayRate <- mcstoc(rnorm, type="U", seed=6824, mean=0.141, sd=0.0224)

RepDPTLDDelay <- RepDDelayedTreat*RepDPTLDDelayRate

RepDnoPTLDDelay <- RepDDelayedTreat-RepDPTLDDelay

#Add the PDLT yes and no groups Together

RepDPTLD <- RepDPTLDonTime+RepDPTLDDelay

RepDNoPTLD <- RepDnoPTLDonTime + RepDnoPTLDDelay

#Cost for the PTLD Group.

RepDPTLDCostRate <- mcstoc(rlnorm, type="V", seed=6824, mean=8.38, sd=0.55)

RepDPTLDCost <- RepDPTLD*RepDPTLDCostRate

#Cost for the non PTLD Group

RepDNoPTLDCostRate <- mcstoc(rlnorm, type="V", seed=6824, mean=6.19, sd=1.38)

RepDNoPTLDCost <- RepDNoPTLD*RepDNoPTLDCostRate

#Societal Cost

SocietyCostRate <- mcstoc(rlnorm, type="V", seed=6824, mean=5.72, sd=1.72)

RepDSocietyCost <- RepDDiagnosed*SocietyCostRate

#Finish it up#

RepDDxResults <- mc(Cases, RepDDiagnosed, RepDNeuroborrel, RepDResidNeuro,

RepDDALYs, RepDDelayedTreat, RepDOnTimeTreat,

RepDPTLDonTime, RepDnoPTLDonTime, RepDPTLDDelay,

RepDnoPTLDDelay, RepDPTLD, RepDNoPTLD, RepDPTLDCost,

RepDNoPTLDCost, RepelProtectRate, RepelProtectCases,

RepDReported, RepDunreported, RepDSocietyCost)

print (RepDDxResults, digits=3)

summary(RepDDxResults)

### Cases and outcomes saved with use of tick repellent###

# For Misdiagnosed #

RepCaseSave <- HiCases - RepelProtectCases

RepMisSave <- HiMisDx - RepMisDx

RepMisCard <- HiCarditis - RepCarditis

RepMisCost <- HiCost - RepCost

RepMisDALY <- HiDALYs - RepDALYs

RepMisNeuro <- HiNeuroborrel - RepNeuroborrel

RepMisNeurResid <- HiResidNeuro - RepResidNeuro

RepMisSocietySave <- HiSocietyCost - RepSocietyCost

# For Diagnosed #

RepDxReported <- HiReported - RepDReported

RepDxUnreported <- Hiunreported - RepDunreported

RepDxSave <- HiDDiagnosed - RepDDiagnosed

RepDxNeuro <- HiDNeuroborrel - RepDNeuroborrel

RepDxNeurResid <- HiDResidNeuro - RepDResidNeuro

RepDxDALY <- HiDDALYs - RepDDALYs

RepDxDelay <- HiDDelayedTreat - RepDDelayedTreat

RepDxTimelyTreat <- HiDOnTimeTreat - RepDOnTimeTreat

RepDxPTLDonTime <- HiDPTLDonTime - RepDPTLDonTime

RepDxNoPTLDonTime <- HiDnoPTLDonTime - RepDnoPTLDonTime

RepDxPTLDDelay <- HiDPTLDDelay - RepDPTLDDelay

RepDxNoPTLDDelay <- HiDnoPTLDDelay - RepDnoPTLDDelay

RepDXPTLDTotSave <- HiDPTLD - RepDPTLD

RepDxNoPTLDTotSave <- HiDNoPTLD - RepDNoPTLD

RepDxPTLDCostSave <- HiDPTLDCost - RepDPTLDCost

RepDxNoPTLDCostSave <- HiDNoPTLDCost - RepDNoPTLDCost

RepDxSocietySave <- HiDSocietyCost - RepDSocietyCost

RepSaveResult <- mc(RepCaseSave, RepMisSave, RepMisCard, RepMisCost,

RepMisDALY, RepMisNeuro, RepMisNeurResid, RepDxSave,

RepDxNeuro, RepDxNeurResid, RepDxDALY, RepDxDelay,

RepDxTimelyTreat, RepDxPTLDonTime, RepDxNoPTLDonTime,

RepDxPTLDDelay, RepDxNoPTLDDelay, RepDXPTLDTotSave,

RepDxNoPTLDTotSave, RepDxPTLDCostSave, RepDxNoPTLDCostSave,

RepDxReported, RepDxUnreported, RepMisSocietySave, RepDxSocietySave)

print (RepSaveResult, digits=3)

summary(RepSaveResult)

###############################################################

####### Below Median Years Only (2014-15, 18-19, 21) ########

###############################################################

### For the Misdiagnosis Side of the Map ###

LoCases <- mcstoc(rnorm, type="V", seed=6824, mean=6776, sd=982.9)

LoMisDx <- LoCases*.72

LoCarditisRate <- mcstoc(runif, type="U", seed=6824, min=0.04, max=0.10)

LoCostRate <- mcstoc(rlnorm, type="U", seed=6824, mean=6.19, sd=1.38)

LoNeuroRate <- mcstoc(rnorm, type="U", seed=6824, mean=0.0884, sd=0.00740)

LoResidNeuroRate <- mcstoc(rnorm, type="U", seed=6824, mean=.2456, sd=.0403)

SocietyCostRate <- mcstoc(rlnorm, type="V", seed=6824, mean=5.72, sd=1.72)

LoSocietyCost <- LoMisDx*SocietyCostRate

LoCarditis <- LoMisDx*LoCarditisRate

LoCost <- LoMisDx*LoCostRate

LoDALYs <- LoMisDx*1.661

LoNeuroborrel <- LoMisDx*LoNeuroRate

LoResidNeuro <-LoNeuroborrel*LoResidNeuroRate

LoMisDxResults <- mc(LoCases, LoMisDx, LoCarditis, LoCost, LoDALYs,

LoNeuroborrel, LoResidNeuro, LoSocietyCost)

print (LoMisDxResults, digits=3)

summary(LoMisDxResults)

### For the Diagnosed and Treated Side of the Map ###

#Estimate the Diagnosed Cases

LoDCases <- mcstoc(rnorm, type="V", seed=6824, mean=6776, sd=982.9)

LoDDiagnosed <- LoDCases*.28

LoReported <- LoDDiagnosed*.374

Lounreported <- LoDDiagnosed - LoReported

#Estimate Neuroborreliosis and residual Neuro

LoDNeuroRate <- mcstoc(rnorm, type="U", seed=6824, mean=0.0884, sd=0.00740)

LoDResidNeuroRate <- mcstoc(rnorm, type="U", seed=6824, mean=.2456, sd=.0403)

LoDNeuroborrel <- LoDDiagnosed*LoDNeuroRate

LoDResidNeuro <-LoDNeuroborrel*LoDResidNeuroRate

#Estimate DALYS

LoDDALYs <- LoDDiagnosed*1.661

#Estimate delayed and on time treatment

LoDDelayRate <- mcstoc(rnorm, type="U", seed=6824, mean=.3098, sd=.01658)

LoDDelayedTreat <- LoDDiagnosed*LoDDelayRate

LoDOnTimeTreat <- LoDDiagnosed-LoDDelayedTreat

##For On Time Treatment##

#Estimate PTLD and No PTLD

LoDPTLDonTimeRate <- mcstoc(rnorm, type="U", seed=6824, mean=0.0733, sd=0.01125)

LoDPTLDonTime <- LoDOnTimeTreat*LoDPTLDonTimeRate

LoDnoPTLDonTime <- LoDOnTimeTreat-LoDPTLDonTime

##For Delay Treatment##

#Estimate PTLD and No PTLD

LoDPTLDDelayRate <- mcstoc(rnorm, type="U", seed=6824, mean=0.141, sd=0.0224)

LoDPTLDDelay <- LoDDelayedTreat*LoDPTLDDelayRate

LoDnoPTLDDelay <- LoDDelayedTreat-LoDPTLDDelay

#Add the PDLT yes and no groups Together

LoDPTLD <- LoDPTLDonTime+LoDPTLDDelay

LoDNoPTLD <- LoDnoPTLDonTime + LoDnoPTLDDelay

#Cost for the PTLD Group.

LoDPTLDCostRate <- mcstoc(rlnorm, type="V", seed=6824, mean=8.38, sd=0.55)

LoDPTLDCost <- LoDPTLD*LoDPTLDCostRate

#Cost for the non PTLD Group

LoDNoPTLDCostRate <- mcstoc(rlnorm, type="V", seed=6824, mean=6.19, sd=1.38)

LoDNoPTLDCost <- LoDNoPTLD*LoDNoPTLDCostRate

#Societal Cost

SocietyCostRate <- mcstoc(rlnorm, type="V", seed=6824, mean=5.72, sd=1.72)

LoDSocietyCost <- LoDDiagnosed*SocietyCostRate

#Finish it up#

LoDDxResults <- mc(LoDCases, LoDDiagnosed, LoReported, Lounreported, LoDNeuroborrel, LoDResidNeuro,

LoDDALYs, LoDDelayedTreat, LoDOnTimeTreat, LoDPTLDonTime,

LoDnoPTLDonTime, LoDPTLDDelay, LoDnoPTLDDelay, LoDPTLD,

LoDNoPTLD, LoDPTLDCost, LoDNoPTLDCost, LoDSocietyCost)

print (LoDDxResults, digits=3)

summary(LoDDxResults)

### Cases and outcomes difference between hi and low years###

# For Misdiagnosed #

LowCaseSave <- HiCases - LoCases

LowMisSave <- HiMisDx - LoMisDx

LowMisCard <- HiCarditis - LoCarditis

LowMisCost <- HiCost - LoCost

LowMisDALY <- HiDALYs - LoDALYs

LowMisNeuro <- HiNeuroborrel - LoNeuroborrel

LowMisNeurResid <- HiResidNeuro - LoResidNeuro

LowMisSocietySave <- HiSocietyCost - LoSocietyCost

# For Diagnosed #

LowDxReported <- HiReported - LoReported

LowDxUnreported <- Hiunreported - Lounreported

LowDxSave <- HiDDiagnosed - LoDDiagnosed

LowDxNeuro <- HiDNeuroborrel - LoDNeuroborrel

LowDxNeurResid <- HiDResidNeuro - LoDResidNeuro

LowDxDALY <- HiDDALYs - LoDDALYs

LowDxDelay <- HiDDelayedTreat - LoDDelayedTreat

LowDxTimelyTreat <- HiDOnTimeTreat - LoDOnTimeTreat

LowDxPTLDonTime <- HiDPTLDonTime - LoDPTLDonTime

LowDxNoPTLDonTime <- HiDnoPTLDonTime - LoDnoPTLDonTime

LowDxPTLDDelay <- HiDPTLDDelay - LoDPTLDDelay

LowDxNoPTLDDelay <- HiDnoPTLDDelay - LoDnoPTLDDelay

LowDXPTLDTotSave <- HiDPTLD - LoDPTLD

LowDxNoPTLDTotSave <- HiDNoPTLD - LoDNoPTLD

LowDxPTLDCostSave <- HiDPTLDCost - LoDPTLDCost

LowDxNoPTLDCostSave <- HiDNoPTLDCost - LoDNoPTLDCost

LowDxSocietySave <- HiDSocietyCost - LoDSocietyCost

TotalLowSocietydif <- LowMisSocietySave + LowDxSocietySave

TotalLowDalyDif <- LowMisDALY + LowDxDALY

TotalPatientCostDif <- LowMisCost + LowDxPTLDCostSave + LowDxNoPTLDCostSave

LowSaveResult <- mc(LowCaseSave, LowMisSave, LowMisCard, LowMisCost,

LowMisDALY, LowMisNeuro, LowMisNeurResid, LowDxSave,

LowDxNeuro, LowDxNeurResid, LowDxDALY, LowDxDelay,

LowDxTimelyTreat, LowDxPTLDonTime, LowDxNoPTLDonTime,

LowDxPTLDDelay, LowDxNoPTLDDelay, LowDXPTLDTotSave,

LowDxNoPTLDTotSave, LowDxPTLDCostSave, LowDxNoPTLDCostSave,

LowDxReported, LowDxUnreported, LowMisSocietySave, LowDxSocietySave,

TotalLowSocietydif, TotalLowDalyDif, TotalPatientCostDif)

print (LowSaveResult, digits=3)

summary(LowSaveResult)

############################################################

################ SENSITIVITY ANALYSIS ####################

############################################################

####### Reported cases -1 SD #######

#Estimate the Diagnosed Cases

Cases <- mcstoc(rnorm, type="V", seed=6824, mean=8206.7, sd=244.3)

Diagnosed <- Cases*.28

Reported <- Diagnosed*.374

unreported <- Diagnosed - Reported

#Estimate delayed and on time treatment

DelayRate <- mcstoc(rnorm, type="U", seed=6824, mean=.3098, sd=.01658)

DelayedTreat <- Diagnosed*DelayRate

OnTimeTreat <- Diagnosed-DelayedTreat

##For On Time Treatment##

#Estimate PTLD and No PTLD

PTLDonTimeRate <- mcstoc(rnorm, type="U", seed=6824, mean=0.0733, sd=0.01125)

PTLDonTime <- OnTimeTreat*PTLDonTimeRate

noPTLDonTime <- OnTimeTreat-PTLDonTime

##For Delay Treatment##

#Estimate PTLD and No PTLD

PTLDDelayRate <- mcstoc(rnorm, type="U", seed=6824, mean=0.141, sd=0.0224)

PTLDDelay <- DelayedTreat*PTLDDelayRate

noPTLDDelay <- DelayedTreat-PTLDDelay

#Add the PDLT yes and no groups Together

PTLD <- PTLDonTime+PTLDDelay

NoPTLD <- noPTLDonTime + noPTLDDelay

#Cost for the PTLD Group.

PTLDCostRate <- mcstoc(rlnorm, type="V", seed=6824, mean=8.38, sd=0.55)

PTLDCost <- PTLD*PTLDCostRate

#Cost for the non PTLD Group

NoPTLDCostRate <- mcstoc(rlnorm, type="V", seed=6824, mean=6.19, sd=1.38)

NoPTLDCost <- NoPTLD*NoPTLDCostRate

TotalCost <- PTLDCost+NoPTLDCost

#Finish it up#

CaseMinusSD <- mc(Cases, DelayedTreat,PTLD, TotalCost)

print (CaseMinusSD, digits=3)

summary(CaseMinusSD)

####### Reported cases +1 SD #######

#Estimate the Diagnosed Cases

Cases <- mcstoc(rnorm, type="V", seed=6824, mean=8695.3, sd=244.3)

Diagnosed <- Cases*.28

Reported <- Diagnosed*.374

unreported <- Diagnosed - Reported

#Estimate delayed and on time treatment

DelayRate <- mcstoc(rnorm, type="U", seed=6824, mean=.3098, sd=.01658)

DelayedTreat <- Diagnosed*DelayRate

OnTimeTreat <- Diagnosed-DelayedTreat

##For On Time Treatment##

#Estimate PTLD and No PTLD

PTLDonTimeRate <- mcstoc(rnorm, type="U", seed=6824, mean=0.0733, sd=0.01125)

PTLDonTime <- OnTimeTreat*PTLDonTimeRate

noPTLDonTime <- OnTimeTreat-PTLDonTime

##For Delay Treatment##

#Estimate PTLD and No PTLD

PTLDDelayRate <- mcstoc(rnorm, type="U", seed=6824, mean=0.141, sd=0.0224)

PTLDDelay <- DelayedTreat*PTLDDelayRate

noPTLDDelay <- DelayedTreat-PTLDDelay

#Add the PDLT yes and no groups Together

PTLD <- PTLDonTime+PTLDDelay

NoPTLD <- noPTLDonTime + noPTLDDelay

#Cost for the PTLD Group.

PTLDCostRate <- mcstoc(rlnorm, type="V", seed=6824, mean=8.38, sd=0.55)

PTLDCost <- PTLD*PTLDCostRate

#Cost for the non PTLD Group

NoPTLDCostRate <- mcstoc(rlnorm, type="V", seed=6824, mean=6.19, sd=1.38)

NoPTLDCost <- NoPTLD*NoPTLDCostRate

TotalCost <- PTLDCost+NoPTLDCost

#Finish it up#

CasePlusSD <- mc(Cases, DelayedTreat,PTLD, TotalCost)

print (CasePlusSD, digits=3)

summary(CasePlusSD)

####### Delayed Treatment -1 SD #######

#Estimate the Diagnosed Cases

Cases <- mcstoc(rnorm, type="V", seed=6824, mean=8451, sd=244.3)

Diagnosed <- Cases*.28

Reported <- Diagnosed*.374

unreported <- Diagnosed - Reported

#Estimate delayed and on time treatment

DelayRate <- mcstoc(rnorm, type="U", seed=6824, mean=.2930, sd=.01658)

DelayedTreat <- Diagnosed*DelayRate

OnTimeTreat <- Diagnosed-DelayedTreat

##For On Time Treatment##

#Estimate PTLD and No PTLD

PTLDonTimeRate <- mcstoc(rnorm, type="U", seed=6824, mean=0.0733, sd=0.01125)

PTLDonTime <- OnTimeTreat*PTLDonTimeRate

noPTLDonTime <- OnTimeTreat-PTLDonTime

##For Delay Treatment##

#Estimate PTLD and No PTLD

PTLDDelayRate <- mcstoc(rnorm, type="U", seed=6824, mean=0.141, sd=0.0224)

PTLDDelay <- DelayedTreat*PTLDDelayRate

noPTLDDelay <- DelayedTreat-PTLDDelay

#Add the PDLT yes and no groups Together

PTLD <- PTLDonTime+PTLDDelay

NoPTLD <- noPTLDonTime + noPTLDDelay

#Cost for the PTLD Group.

PTLDCostRate <- mcstoc(rlnorm, type="V", seed=6824, mean=8.38, sd=0.55)

PTLDCost <- PTLD*PTLDCostRate

#Cost for the non PTLD Group

NoPTLDCostRate <- mcstoc(rlnorm, type="V", seed=6824, mean=6.19, sd=1.38)

NoPTLDCost <- NoPTLD*NoPTLDCostRate

TotalCost <- PTLDCost+NoPTLDCost

#Finish it up#

DelayMinusSD <- mc(Cases, DelayedTreat,PTLD, TotalCost)

print (DelayMinusSD, digits=3)

summary(DelayMinusSD)

####### Delayed Treatment +1 SD #######

#Estimate the Diagnosed Cases

Cases <- mcstoc(rnorm, type="V", seed=6824, mean=8451, sd=244.3)

Diagnosed <- Cases*.28

Reported <- Diagnosed*.374

unreported <- Diagnosed - Reported

#Estimate delayed and on time treatment

DelayRate <- mcstoc(rnorm, type="U", seed=6824, mean=.3098, sd=.01658)

DelayedTreat <- Diagnosed*DelayRate

OnTimeTreat <- Diagnosed-DelayedTreat

##For On Time Treatment##

#Estimate PTLD and No PTLD

PTLDonTimeRate <- mcstoc(rnorm, type="U", seed=6824, mean=0.0733, sd=0.01125)

PTLDonTime <- OnTimeTreat*PTLDonTimeRate

noPTLDonTime <- OnTimeTreat-PTLDonTime

##For Delay Treatment##

#Estimate PTLD and No PTLD

PTLDDelayRate <- mcstoc(rnorm, type="U", seed=6824, mean=0.141, sd=0.0224)

PTLDDelay <- DelayedTreat*PTLDDelayRate

noPTLDDelay <- DelayedTreat-PTLDDelay

#Add the PDLT yes and no groups Together

PTLD <- PTLDonTime+PTLDDelay

NoPTLD <- noPTLDonTime + noPTLDDelay

#Cost for the PTLD Group.

PTLDCostRate <- mcstoc(rlnorm, type="V", seed=6824, mean=8.38, sd=0.55)

PTLDCost <- PTLD*PTLDCostRate

#Cost for the non PTLD Group

NoPTLDCostRate <- mcstoc(rlnorm, type="V", seed=6824, mean=6.19, sd=1.38)

NoPTLDCost <- NoPTLD*NoPTLDCostRate

TotalCost <- PTLDCost+NoPTLDCost

#Finish it up#

DelayPlusSD <- mc(Cases, DelayedTreat,PTLD, TotalCost)

print (DelayPlusSD, digits=3)

summary(DelayPlusSD)

####### PTLDS -1 SD #######

#Estimate the Diagnosed Cases

Cases <- mcstoc(rnorm, type="V", seed=6824, mean=8451, sd=244.3)

Diagnosed <- Cases*.28

Reported <- Diagnosed*.374

unreported <- Diagnosed - Reported

#Estimate delayed and on time treatment

DelayRate <- mcstoc(rnorm, type="U", seed=6824, mean=.3262, sd=.01658)

DelayedTreat <- Diagnosed*DelayRate

OnTimeTreat <- Diagnosed-DelayedTreat

##For On Time Treatment##

#Estimate PTLD and No PTLD

PTLDonTimeRate <- mcstoc(rnorm, type="U", seed=6824, mean=0.0621, sd=0.01125)

PTLDonTime <- OnTimeTreat*PTLDonTimeRate

noPTLDonTime <- OnTimeTreat-PTLDonTime

##For Delay Treatment##

#Estimate PTLD and No PTLD

PTLDDelayRate <- mcstoc(rnorm, type="U", seed=6824, mean=0.119, sd=0.0224)

PTLDDelay <- DelayedTreat*PTLDDelayRate

noPTLDDelay <- DelayedTreat-PTLDDelay

#Add the PDLT yes and no groups Together

PTLD <- PTLDonTime+PTLDDelay

NoPTLD <- noPTLDonTime + noPTLDDelay

#Cost for the PTLD Group.

PTLDCostRate <- mcstoc(rlnorm, type="V", seed=6824, mean=8.38, sd=0.55)

PTLDCost <- PTLD*PTLDCostRate

#Cost for the non PTLD Group

NoPTLDCostRate <- mcstoc(rlnorm, type="V", seed=6824, mean=6.19, sd=1.38)

NoPTLDCost <- NoPTLD*NoPTLDCostRate

TotalCost <- PTLDCost+NoPTLDCost

#Finish it up#

PTLDSMinusSD <- mc(Cases, DelayedTreat,PTLD, TotalCost)

print (PTLDSMinusSD, digits=3)

summary(PTLDSMinusSD)

####### PTLDS +1 SD #######

#Estimate the Diagnosed Cases

Cases <- mcstoc(rnorm, type="V", seed=6824, mean=8451, sd=244.3)

Diagnosed <- Cases*.28

Reported <- Diagnosed*.374

unreported <- Diagnosed - Reported

#Estimate delayed and on time treatment

DelayRate <- mcstoc(rnorm, type="U", seed=6824, mean=.3262, sd=.01658)

DelayedTreat <- Diagnosed*DelayRate

OnTimeTreat <- Diagnosed-DelayedTreat

##For On Time Treatment##

#Estimate PTLD and No PTLD

PTLDonTimeRate <- mcstoc(rnorm, type="U", seed=6824, mean=0.0846, sd=0.01125)

PTLDonTime <- OnTimeTreat*PTLDonTimeRate

noPTLDonTime <- OnTimeTreat-PTLDonTime

##For Delay Treatment##

#Estimate PTLD and No PTLD

PTLDDelayRate <- mcstoc(rnorm, type="U", seed=6824, mean=0.1634, sd=0.0224)

PTLDDelay <- DelayedTreat*PTLDDelayRate

noPTLDDelay <- DelayedTreat-PTLDDelay

#Add the PDLT yes and no groups Together

PTLD <- PTLDonTime+PTLDDelay

NoPTLD <- noPTLDonTime + noPTLDDelay

#Cost for the PTLD Group.

PTLDCostRate <- mcstoc(rlnorm, type="V", seed=6824, mean=8.38, sd=0.55)

PTLDCost <- PTLD*PTLDCostRate

#Cost for the non PTLD Group

NoPTLDCostRate <- mcstoc(rlnorm, type="V", seed=6824, mean=6.19, sd=1.38)

NoPTLDCost <- NoPTLD*NoPTLDCostRate

TotalCost <- PTLDCost+NoPTLDCost

#Finish it up#

PTLDSPlusSD <- mc(Cases, DelayedTreat,PTLD, TotalCost)

print (PTLDSPlusSD, digits=3)

summary(PTLDSPlusSD)

####### Cost -1 SD #######

#Estimate the Diagnosed Cases

Cases <- mcstoc(rnorm, type="V", seed=6824, mean=8451, sd=244.3)

Diagnosed <- Cases*.28

Reported <- Diagnosed*.374

unreported <- Diagnosed - Reported

#Estimate delayed and on time treatment

DelayRate <- mcstoc(rnorm, type="U", seed=6824, mean=.3262, sd=.01658)

DelayedTreat <- Diagnosed*DelayRate

OnTimeTreat <- Diagnosed-DelayedTreat

##For On Time Treatment##

#Estimate PTLD and No PTLD

PTLDonTimeRate <- mcstoc(rnorm, type="U", seed=6824, mean=0.0733, sd=0.01125)

PTLDonTime <- OnTimeTreat*PTLDonTimeRate

noPTLDonTime <- OnTimeTreat-PTLDonTime

##For Delay Treatment##

#Estimate PTLD and No PTLD

PTLDDelayRate <- mcstoc(rnorm, type="U", seed=6824, mean=0.141, sd=0.0224)

PTLDDelay <- DelayedTreat*PTLDDelayRate

noPTLDDelay <- DelayedTreat-PTLDDelay

#Add the PDLT yes and no groups Together

PTLD <- PTLDonTime+PTLDDelay

NoPTLD <- noPTLDonTime + noPTLDDelay

#Cost for the PTLD Group.

PTLDCostRate <- mcstoc(rlnorm, type="V", seed=6824, mean=7.398, sd=0.933)

PTLDCost <- PTLD*PTLDCostRate

#Cost for the non PTLD Group

NoPTLDCostRate <- mcstoc(rlnorm, type="V", seed=6824, mean=4.86, sd=1.77)

NoPTLDCost <- NoPTLD*NoPTLDCostRate

TotalCost <- PTLDCost+NoPTLDCost

#Finish it up#

CostMinusSD <- mc(Cases, DelayedTreat,PTLD, TotalCost)

print (CostMinusSD, digits=3)

summary(CostMinusSD)

####### Cost +1 SD #######

#Estimate the Diagnosed Cases

Cases <- mcstoc(rnorm, type="V", seed=6824, mean=8451, sd=244.3)

Diagnosed <- Cases*.28

Reported <- Diagnosed*.374

unreported <- Diagnosed - Reported

#Estimate delayed and on time treatment

DelayRate <- mcstoc(rnorm, type="U", seed=6824, mean=.3262, sd=.01658)

DelayedTreat <- Diagnosed*DelayRate

OnTimeTreat <- Diagnosed-DelayedTreat

##For On Time Treatment##

#Estimate PTLD and No PTLD

PTLDonTimeRate <- mcstoc(rnorm, type="U", seed=6824, mean=0.0733, sd=0.01125)

PTLDonTime <- OnTimeTreat*PTLDonTimeRate

noPTLDonTime <- OnTimeTreat-PTLDonTime

##For Delay Treatment##

#Estimate PTLD and No PTLD

PTLDDelayRate <- mcstoc(rnorm, type="U", seed=6824, mean=0.141, sd=0.0224)

PTLDDelay <- DelayedTreat*PTLDDelayRate

noPTLDDelay <- DelayedTreat-PTLDDelay

#Add the PDLT yes and no groups Together

PTLD <- PTLDonTime+PTLDDelay

NoPTLD <- noPTLDonTime + noPTLDDelay

#Cost for the PTLD Group.

PTLDCostRate <- mcstoc(rlnorm, type="V", seed=6824, mean=8.859, sd=0.378)

PTLDCost <- PTLD*PTLDCostRate

#Cost for the non PTLD Group

NoPTLDCostRate <- mcstoc(rlnorm, type="V", seed=6824, mean=6.91, sd=1.12)

NoPTLDCost <- NoPTLD*NoPTLDCostRate

TotalCost <- PTLDCost+NoPTLDCost

#Finish it up#

CostPlusSD <- mc(Cases, DelayedTreat,PTLD, TotalCost)

print (CostPlusSD, digits=3)

summary(CostPlusSD)
